# Balancing Thrombosis and Bleeding: A Case of Massive Haemothorax Complicating Anticoagulation for Pulmonary Embolism

**DOI:** 10.7759/cureus.111428

**Published:** 2026-06-24

**Authors:** Essam Arabi, Mohamed Elobaid, Ahmed I Idriss, Ahmed Sayedahmed, Haytham Makki, Mohamed M Mabuie

**Affiliations:** 1 General Internal Medicine, University Hospital Kerry, Tralee, IRL; 2 Surgery, University of Khartoum, Khartoum, SDN; 3 General Surgery, Cavan General Hospital, Cavan, IRL; 4 Internal Medicine, Faculty of Medicine, University of Khartoum, Khartoum, SDN; 5 Internal Medicine, University Hospital Kerry, Tralee, IRL; 6 Internal Medicine, Omdurman Islamic University, Omdurman, SDN; 7 Cardiology, Bon Secours Hospital, Tralee, IRL; 8 Surgery, Faculty of Medicine, University of Khartoum, Khartoum, SDN

**Keywords:** anticoagulation, bleeding complications, delayed haemothorax, haemothorax, massive haemothorax, pleural hemorrhage, pulmonary embolism, thoracic trauma

## Abstract

Massive haemothorax is a life-threatening condition most commonly associated with thoracic trauma, malignancy, or iatrogenic injury. Anticoagulation, while essential for thromboembolic disease management, may precipitate or exacerbate occult bleeding complications. We report the case of a 69-year-old woman who presented with pleuritic chest pain, productive cough, and progressive dyspnoea following a recent fall and long-haul travel. CT pulmonary angiography confirmed segmental and subsegmental pulmonary emboli with bilateral consolidation, and therapeutic low-molecular-weight heparin was initiated. On the third hospital day, she developed sudden hypoxia, left-sided chest pain, and a significant haemoglobin decline. Repeat imaging demonstrated a massive left haemothorax with lung collapse and mediastinal shift, together with previously unrecognised right-sided rib fractures. Anticoagulation was immediately discontinued and reversed, and the patient underwent blood transfusion and chest drainage, yielding over 2.4 L of blood. Residual loculated haemothorax was successfully managed using image-guided catheter drainage without surgical intervention. This case highlights the rare occurrence of delayed contralateral haemothorax complicating anticoagulation and underscores the importance of recognising atypical bleeding presentations and balancing thrombotic and haemorrhagic risk through multidisciplinary decision-making.

## Introduction

Haemothorax most commonly arises from thoracic trauma, intercostal vessel injury, malignancy, or iatrogenic procedures [1,2]. Anticoagulation may unmask or exacerbate small pleural or vascular injuries, resulting in significant intrathoracic bleeding [3-5]. Massive haemothorax is generally defined as an initial pleural blood loss exceeding 1.5 L or ongoing bleeding greater than 200 mL/hour [6].

Contralateral haemothorax following unilateral thoracic trauma is uncommon but recognised and is thought to result from transmission of mechanical forces causing vascular or parenchymal injury opposite to the site of impact [7,8].

Management of massive haemothorax requires prompt recognition, haemodynamic stabilisation, cessation of anticoagulation, and pleural drainage [1,6]. Decisions regarding resumption of anticoagulation following life-threatening bleeding remain challenging and require careful individualised assessment of thrombotic and haemorrhagic risk [9,10].

## Case presentation

A 69-year-old woman presented with a one-week history of right-sided pleuritic chest pain, productive cough, and progressively worsening shortness of breath. She reported a recent mechanical fall onto her right torso while abroad, followed by a long-haul flight home, both recognised risk factors for venous thromboembolism [4,5]. She had also been treated with clarithromycin for a lower respiratory tract infection. Her medical history included hypertension and chronic sinusitis.

On initial assessment, her blood pressure was 158/84 mmHg, heart rate was 93 bpm, temperature was 36.1°C, and oxygen saturation was 93% on room air. Clinical examination demonstrated reduced air entry at the left lung base. Laboratory investigations revealed a white blood cell count of 16×10^9^/L, neutrophils 14×10^9^/L, C-reactive protein 83 mg/L, and D-dimer 3.39 mg/L (Tables 1, 2). Chest radiography demonstrated left lower lobe atelectasis and a small pleural effusion (Figure 1).

**Table 1:** Initial hematological and coagulation investigations demonstrating leukocytosis, neutrophilia, and elevated D-dimer levels at presentation, supporting an inflammatory process and raising suspicion for venous thromboembolism. WBC: white blood cell count; RBC: red blood cell count; Hb: haemoglobin; HCT: haematocrit; MCV: mean corpuscular volume; MCH: mean corpuscular haemoglobin; MCHC: mean corpuscular haemoglobin concentration; RDW: red cell distribution width; PLT: platelet count; PT: prothrombin time; APTT: activated partial thromboplastin time.

Test	Result	Units	Reference range
WBC	16.1	x10^9^/L	4.4-11.3
RBC	4.38	x10^12^/L	3.9-5.3
HB	12.7	g/dL	11.7-15.9
HCT	0.389	L/L	0.35-0.46
MCV	88.8	fL	80-96
MCH	29.0	pg	26.0-34.0
MCHC	32.6	g/dL	31.0-37.0
RDW	13.2	%	11.6-15
PLT	188	x10^9^/L	140-440
Neutrophils	14.12	x10^9^/L	1.4-6.6
Lymphocytes	1.16	x10^9^/L	0.9-3.2
Monocytes	0.83	x10^9^/L	0.15-1.3
Eosinophils	0.00	x10^9^/L	0.04-0.4
Basophils	0.03	x10^9^/L	0-0.1
PT	11.7	sec	9.7-11.3
APTT	27.5	sec	21–29
D-Dimer	3.39	mg/L FEU	0-0.5

**Table 2:** Initial biochemical investigations demonstrating preserved renal and hepatic function at presentation, with no significant laboratory evidence of organ dysfunction. ALT: alanine aminotransferase; AST: aspartate aminotransferase; alkaline phosphatase; GGT: gamma-glutamyl transferase; bilirubin, total serum bilirubin.

Test	Result	Units	Reference range
Sodium	138	mmol/L	133-146
Potassium	3.8	mmol/L	3.5-5.3
Chloride	102	mmol/L	95-108
Urea	4.8	mmol/L	2.5-7.8
Creatinine	58	µmol/L	49-90
Total protein	70	g/L	60-80
Albumin	41	g/L	35-50
AST	13	U/L	1-35
ALT	18	U/L	1-35
Alkaline phosphatase	75	U/L	40-120
GGT	17	U/L	1-38
Total bilirubin	23	µmol/L	5-21
Calcium	2.32	mmol/L	2.20-2.65
Phosphate	1.18	mmol/L	0.80-1.50
Magnesium	0.87	mmol/L	0.70-1.00
C-reactive protein	83.0	mg/L	0.2-5.0

**Figure 1:**
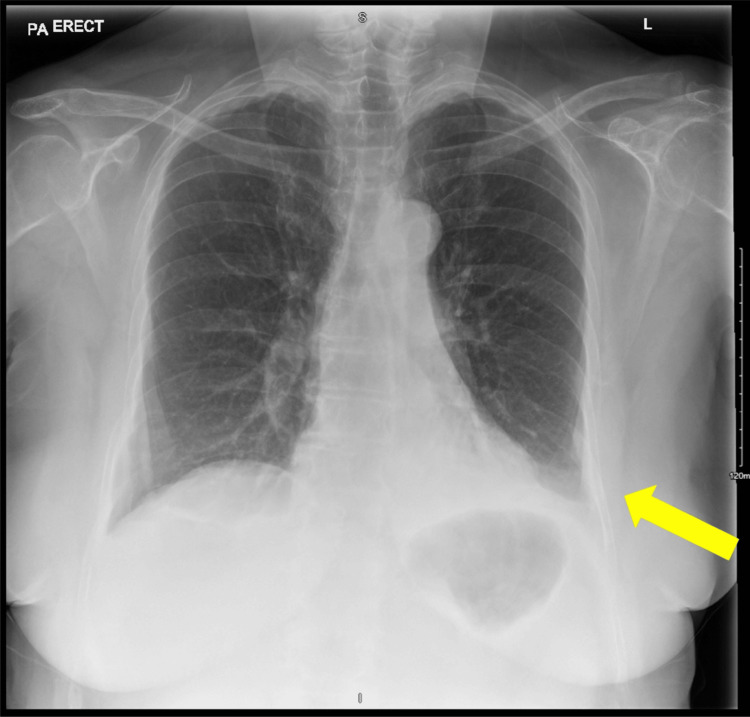
Admission chest radiograph demonstrating left lower lobe atelectatic changes with a small left pleural effusion (arrow). No pneumothorax or definite rib fracture is identified.

Given the elevated D-dimer and relevant clinical risk factors, CT pulmonary angiography (CTPA) was performed, which confirmed segmental and subsegmental pulmonary emboli [4,5], along with bilateral consolidation consistent with infection [9]. Therapeutic low-molecular-weight heparin (LMWH) and antibiotics were commenced in accordance with guideline-based management of pulmonary embolism and pneumonia [4,5,9] (Figure 2).

**Figure 2:**
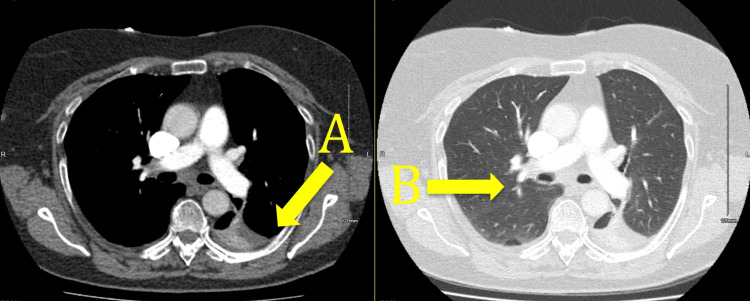
CT pulmonary angiography demonstrating bilateral consolidation and left lower lobe atelectatic changes, more obvious on the left lower lobe (arrow A) with segmental and subsegmental pulmonary emboli (arrow B).

On day three of admission, the patient developed worsening hypoxia requiring high-flow oxygen therapy. Shortly thereafter, she experienced sudden left-sided chest pain accompanied by a haemoglobin drop from 12.7 g/dL on admission to 8 g/dL (Table 3). Repeat CT imaging demonstrated a massive left haemothorax with near-complete lung collapse and mediastinal shift, in addition to previously undetected right-sided rib fractures, recognised causes of delayed haemothorax [7,8] (Figure 3).

**Table 3:** Serial hematological investigations obtained following the development of massive left haemothorax. Results demonstrate persistent anaemia, with a nadir haemoglobin of 7.9 g/dL and reduced haematocrit and red blood cell count, consistent with acute blood loss. Subsequent laboratory trends show gradual stabilisation following blood transfusion, pleural drainage, and supportive management. WBC: white blood cell count; RBC: red blood cell count; Hb: haemoglobin; HCT: haematocrit; MCV: mean corpuscular volume; MCH: mean corpuscular haemoglobin; MCHC: mean corpuscular haemoglobin concentration; RDW: red cell distribution width; PLT: platelet count; PT: prothrombin time; INR: international normalised ratio; APTT: activated partial thromboplastin time.

Test	Day 3	Day 4 (06:43 am)	Day 4 (2:22 pm)	Day 5	Day 6	Day 7	Unit	Reference range
WBC	7.3	5.8	6.8	6.1	6.4	5.7	×10⁹/L	4.4-11.3
RBC	2.63	2.63	2.84	3.22	3.12	3.13	×10¹²/L	3.9-5.3
HB	8.0	7.9	8.4	9.4	9.2	9.0	g/dL	11.7-15.9
HCT	0.234	0.238	0.254	0.284	0.275	0.276	L/L	0.35-0.46
MCV	89.0	90.5	89.4	88.2	88.1	88.2	fL	80-96
MCH	30.4	30.0	29.6	29.2	29.5	28.8	pg	26.0-34.0
MCHC	34.2	33.2	33.1	33.1	33.5	32.6	g/dL	31.0-37.0
RDW	14.9	15.0	14.8	15.7	15.5	15.1	%	11.6-15
PLT	300	346	378	387	406	415	×10⁹/L	140-440
PT	10.7	11.2		10.9	11.0	10.9	seconds	9.7-11.3
INR	1.0	1.1			1.0	1.0		0.8-1.1
APTT	23.4	25.1		25.0	24.9	25.6	seconds	21-29

**Figure 3:**
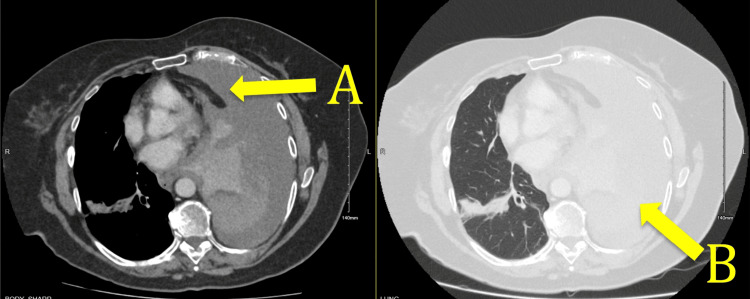
Follow-up CT imaging demonstrating near-complete collapse of the left lung (arrow A) and a massive left-sided haemothorax occupying most of the left hemithorax (arrow B), with associated rightward mediastinal shift.

LMWH was immediately discontinued and reversed with protamine in accordance with haemothorax management principles. The patient required a blood transfusion and chest drain insertion, which yielded more than 2.4 L of blood within 24 hours, fulfilling criteria for massive haemothorax [1].

Management remained conservative with close cardiothoracic surgical input, as no radiological evidence of ongoing active bleeding was identified [1,3]. A residual loculated haemothorax subsequently developed and was successfully treated using image-guided small-bore catheter drainage, an established minimally invasive strategy in stable patients (Figure 4).

**Figure 4:**
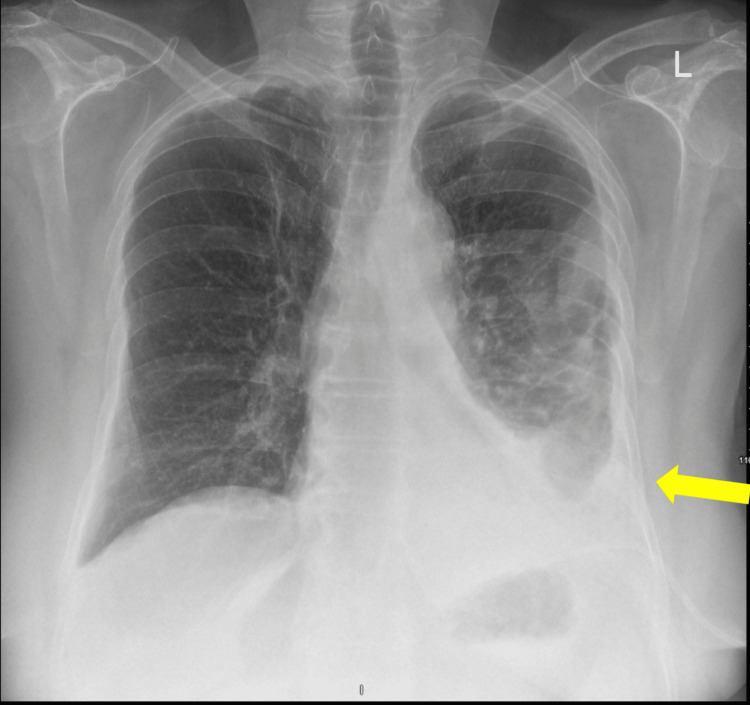
Follow-up chest radiograph demonstrating residual loculated left haemothorax (arrow), with basal percutaneous pigtail catheter in situ.

Repeat CTPA demonstrated no residual pulmonary embolism. Following multidisciplinary discussion, anticoagulation was withheld because of the life-threatening haemorrhage and persistent bleeding risk, consistent with guideline-supported individualised decision-making [4,5].

## Discussion

This case highlights the challenging balance between thrombotic and haemorrhagic risk in patients requiring anticoagulation. Although anticoagulation remains the cornerstone of pulmonary embolism treatment [4,5], it carries a recognised risk of major bleeding, including uncommon complications such as haemothorax [10-12].

Haemothorax is most frequently associated with thoracic trauma and usually occurs ipsilateral to the site of injury due to direct vascular or pulmonary damage [3,6]. However, delayed haemothorax is increasingly recognised and may develop days to weeks after trauma, even when initial imaging findings are unremarkable [7,8].

The contralateral presentation in this case is particularly unusual but has been described previously. Proposed mechanisms include transmission of mechanical forces across the thoracic cage, resulting in occult vascular injury opposite the site of trauma [2,7]. Such injuries may remain clinically silent until anticoagulation precipitates clinically significant bleeding [10-12].

Concurrent pneumonia may also have contributed through pleural inflammation and increased vascular fragility, further predisposing to haemorrhage [9]. The combination of trauma, inflammation, and anticoagulation, therefore, created a high-risk setting for major bleeding.

Management followed established principles, including prompt cessation and reversal of anticoagulation, haemodynamic support, and pleural drainage [1,3]. Surgical intervention is generally reserved for ongoing haemorrhage or failure of conservative management [1]. Development of a residual loculated haemothorax is a recognised complication, and minimally invasive drainage approaches have shown good outcomes in appropriately selected patients [13,14].

A major clinical challenge in this case was determining whether to resume anticoagulation. Current guidelines acknowledge that anticoagulation may be withheld in selected patients, particularly those with subsegmental pulmonary embolism and prohibitive bleeding risk [4,5]. In this patient, the severity of haemorrhage, absence of residual thrombosis, and ongoing bleeding risk supported permanent discontinuation.

## Conclusions

This case illustrates a rare but clinically significant complication of anticoagulation: massive contralateral haemothorax following recent thoracic trauma. Although anticoagulation remains fundamental in the management of pulmonary embolism, it may unmask occult pleural or vascular injuries and precipitate life-threatening bleeding complications. Delayed haemothorax can develop days to weeks after thoracic trauma despite initially unremarkable imaging findings, and contralateral haemothorax, although uncommon, should remain an important diagnostic consideration in patients with a history of chest trauma.

Prompt recognition, early haemodynamic stabilisation, anticoagulation reversal, blood transfusion, and pleural drainage are essential principles of haemothorax management. The successful conservative management in this case, including image-guided drainage of residual loculated haemothorax, further highlights the value of multidisciplinary collaboration and careful patient selection when considering invasive intervention.

This case also emphasises the complexity of anticoagulation decision-making in patients with competing thrombotic and haemorrhagic risks. In selected patients, particularly those with subsegmental pulmonary embolism and extreme bleeding risk, anticoagulation may require withholding following multidisciplinary assessment and individualised risk-benefit evaluation. Recognition of delayed and atypical bleeding presentations is therefore essential to optimise outcomes and minimise avoidable morbidity.
